# Two Independent Functions of Collier/Early B Cell Factor in the Control of *Drosophila* Blood Cell Homeostasis

**DOI:** 10.1371/journal.pone.0148978

**Published:** 2016-02-11

**Authors:** Justine Oyallon, Nathalie Vanzo, Joanna Krzemień, Ismaël Morin-Poulard, Alain Vincent, Michèle Crozatier

**Affiliations:** Centre de Biologie du Développement (CBD), Centre de Biologie Intégrative (CBI), University Paul Sabatier (UPS), CNRS, 118 Route de Narbonne 31062 Toulouse cedex 09, France; Uppsala University, SWEDEN

## Abstract

Blood cell production in the *Drosophila* hematopoietic organ, the lymph gland, is controlled by intrinsic factors and extrinsic signals. Initial analysis of Collier/Early B Cell Factor function in the lymph gland revealed the role of the Posterior Signaling Center (PSC) in mounting a dedicated cellular immune response to wasp parasitism. Further, premature blood cell differentiation when PSC specification or signaling was impaired, led to assigning the PSC a role equivalent to the vertebrate hematopoietic niche. We report here that Collier is expressed in a core population of lymph gland progenitors and cell autonomously maintains this population. The PSC contributes to lymph gland homeostasis by regulating blood cell differentiation, rather than by maintaining core progenitors. In addition to PSC signaling, switching off Collier expression in progenitors is required for efficient immune response to parasitism. Our data show that two independent sites of Collier/Early B Cell Factor expression, hematopoietic progenitors and the PSC, achieve control of hematopoiesis.

## Introduction

Understanding how the balance between progenitor cell maintenance and differentiation is regulated in developing and adult tissues is a major area of investigation. The *Drosophila* larval hematopoietic organ, the lymph gland, is one established model to study the signalling mechanisms controlling this balance, under normal developmental and immunological challenging conditions [1–7]. Under normal developmental conditions, two types of blood cells (called hemocytes) differentiate in the lymph gland, the plasmatocytes endowed with phagocytic activity, and crystal cells required for melanisation. They are released into the circulation, the hemolymph, upon lymph gland dispersal at the onset of metamorphosis [8, 9]. If the larva falls prey to wasp parasitism (i.e. wasps lay eggs in *Drosophila* larvae), a third hemocyte type, the lamellocytes, massively differentiate at the expense of progenitor maintenance, thus provoking lymph gland dispersal before metamorphosis [8, 10, 11]. Lamellocytes released into the hemolymph contribute to encapsulation and neutralisation of parasitoid wasp eggs [8, 12].

The lymph gland is specified in embryos and grows throughout larval development [10, 13, 14]. In third instar larvae, it is composed of a pair of anterior (primary) lobes which contains both progenitors and differentiated hemocytes, and posterior (secondary) lobes which only contain progenitors [5, 8]. Hematopoietic progenitors are located in the inner part of primary lobes, the so-called medullary zone (MZ). Differentiated hemocytes form the cortical zone (CZ) [5, 11, 15]. An intermediate zone (IZ), where postulated intermediate progenitors and committed hemocyte precursors progress towards differentiation is morphologically and molecularly less well defined [5, 11, 15, 16].

The PSC (Posterior Signaling Center), a small cluster of cells located at the posterior tip of primary lobes, is specified by the maintenance of high expression levels of *collier (col)/knot(kn)*, the ortholog of mammalian Early B-cell Factors (EBF), under the control of the homeotic protein Antennapedia (Antp) [10, 13, 17]. Two independent studies proposed that in the larval lymph gland, the PSC plays a role equivalent to the hematopoietic niche of the vertebrate bone marrow [11, 13]. One critical observation was the increased hemocyte differentiation starting in early third instar larvae, in *col* mutant lymph glands [11]. A similar increased differentiation phenotype was observed both in *Antp* and *hedgehog* (*hh)*^*ts*^ mutant lymph glands, and upon specific inactivation of Hh signaling in the medullary zone [13]. Another ligand secreted by PSC cells, PDGF and VEGF-related factor 1 (Pvf1) regulates hemocyte homeostasis in the lymph gland [18, 19]. Finally, genetically modifying the number of PSC cells altered the balance between progenitor maintenance and hemocyte differentiation [13, 20–26]. Together, these observations led to the conclusion that PSC cells act in a non cell autonomous manner to maintain hematopoietic progenitors in the medullary zone. Beside this role in normal developmental conditions, the PSC was shown to be required for massive lamellocyte differentiation either in the lymph gland or in circulation upon wasp parasitism [10, 11, 27]

Detection of low levels of *col* expression in the medullary zone of early third instar larvae raised, however, the question of its potential function during larval hematopoiesis [28]. We report here that *col* is expressed in a subset of medullary zone progenitors and defines a core population of hemocyte progenitors. Removing Col activity specifically from these cells results in loss of medullary zone progenitors and massive hemocyte differentiation in third instar larvae. Similar conclusions were reported in a recent article by Benmimoun et al. (2015), using similar sets of experiments, and starting from the same observation [28]. Besides, either decreasing the number of PSC cells, or altering their signaling properties deregulates hemocyte differentiation in the lymph gland, without loss of core progenitors. Altogether, our data indicate that two independent sites of Col expression in the lymph gland, in core progenitors and in the PSC, control hematopoiesis thus shifting the previously established lymph gland homeostasis paradigm.

## Materials and Methods

### *Drosophila* strains

The following strains were used: *w*^*118*^ (control), *pcol-Gal4*;*UAS-mCD8-GFP* (*pcol>GFP)* [11], PG125 *dome-gal4* (*dome>*) [29], *Cg25C-GFP* [30], *hhF4fGFP* [31], *UAS-col RNAi* [20], *UAS-srp RNAi* (GD12779 Vienna *Drosophila* RNAi Center), *UAS-Myc* and *UAS-TCF*^*DN*^ [20], *UAS-reaper* and *UAS-Dicer2* (Bloomington Stock Center). Larvae were raised, in uncrowded conditions from eggs collected for 8 or 12 hours, at 27°C except for RNAi interference experiments which were performed at 29°C. *UAS-Dicer2* was co-expressed with RNAi constructs. Larvae were developmentally staged at 29°C as follows: early third larval instar (EL3): 66H after egg laying (AEL); mid third larval instar (ML3): 85H AEL, late third larval instar (LL3): 105H AEL.

### Molecular cloning

*domeMESOGFP* transgenic flies were generated as followed: The *domeMESO* sequence was subcloned from *pCasHs43DomeMESO-LacZ* [32] into pS3aG (gift from Thomas Williams, Addgene plasmid # 31171) using the BamH1 and EcoR1 cloning sites. The resulting pS3aG*domeMESO-GFP* plasmid was used to generate *domeMESOGFP* flies using the attP/attB technology [33]. Two independent *Drosophila* lines were created by integration at the *attP*-51B (II) and *attP*-68A4 (III) sites. *domeMESOGFP*-positive cells overlap with *domeless-Gal4>GFP* progenitors in lymph gland anterior lobes.

### Tissue dissection, *in situ* hybridization and antibody staining

Lymph glands were dissected and processed as previously described [11]. The following primary antibodies were used: mouse anti-Col [10]; anti-proPO (1/200; T. Trenczek, Justus-Liebig-University Giessen, Giessen, Germany); anti-P1 (1/30; I. Ando, Institute of Genetics, Biological Research Center of the Hungarian Academy of Science, Szeged, Hungary); anti-Antp (1/100) and anti-Hindsight (Hnt)/pebbled (peb) (1/100) (Developmental Studies Hybridoma Bank). Rabbit anti-H3P (1/200; Upstate Biotechnology); anti-αPS4 (1/200, [11]. For *in situ* hybridization, DIG-labeled antisense RNA probes against *col* [10], *tep4* [11] and *latran (lat)/eye transformer (ey)* [34] were used. *In situ* hybridized samples were mounted in 1XPBS-60% glycerol. Immunostained samples were mounted in Vectashield medium (Vector Laboratories) and analyzed with laser scanning confocal microscopy (Leica SP5). Cell nuclei were visualized using the dye TOPRO3 (Molecular Probes).

### Measurement of crystal cell, plasmatocyte and mitotic indexes

Optimized confocal sections were performed with Leica SP5 for 3D reconstructions. All quantifications were performed using the Volocity software and are representative of at least three independent experiments. Statistical analyses t test (Mann—Whitney nonparametric test) was performed using GraphPad Prism 5 software. Crystal cell and plasmatocyte indexes were displayed as the number of proPO- or Hnt-positive cells and the volume of P1 staining, per primary lobe relative to the lobe’s volume. A minimum of 20 lymph glands were scored per genotype.

### Wasp parasitism

Early third instar larvae were subjected to 1 H of egg-laying by wasps. The non-immune suppressive line of the wasp *Leptopilina boulardi* (NIS or G486 strain) was used [35]. For the lymph gland dispersal assay, larvae were dissected at the appropriate time, without prior fixation and lymph glands were analyzed for anterior lobe integrity. Anterior lobes showing no hemocyte dispersal were considered as intact anterior lobes. Un-parasitized or over-parasitized larvae were excluded from quantifications.

## Results

### Low levels of *col* expression in lymph gland anterior lobes define a sub-medullar zone

**Fig 1:**
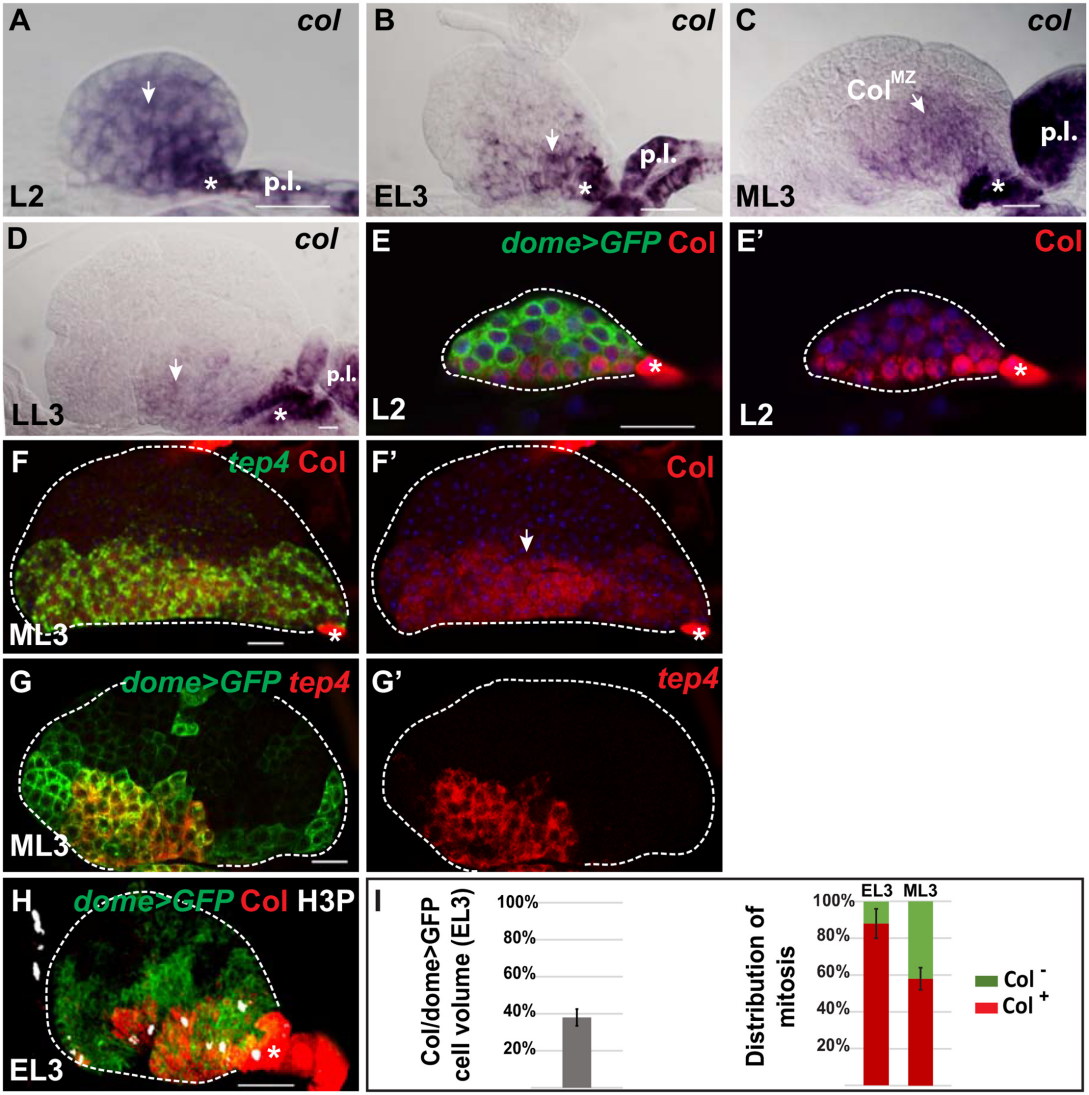
*col* expression in blood cell progenitors during lymph gland development. In this, and subsequent figures, primary lobes of lymph glands are oriented anterior at the left. (A-D) *in situ* hybridization to *col* RNA at different stages of wild type lymph gland development, indicated on each panel. Low level *col* mRNA is detected in the medullary zone (white arrow), and high levels in the PSC (asterisk) and posterior/secondary lobes (p.l.). (E) Immunostaining of *dome>UAS-mCD8GFP* (*dome>GFP*) second instar (L2) lymph glands for Col (red) and GFP (green). TOPRO3 (blue) marks nuclei; (E’) Only Col and TOPRO3 are shown. (F-G’) Single confocal sections. In mid third larval instar (ML3) lymph glands, Col expression (red in F, F’) overlaps *tep4* RNA (green). TOPRO3 (blue) marks nuclei. (G, G’) *tep4* RNA (red) is restricted to a subset of *dome>UAS-mCD8GFP* cells (green). (H) Cell divisions in early third larval instar (EL3) lymph glands, visualized by H3P staining (white). Confocal projection. Col expression (red) is restricted to the innermost part of the medullary zone (green). Most medullary zone mitoses lie inside the Col-positive domain. (I) Left, quantification of the volume of *col*-positive cells (38 ± 9%) relative to total medullary zone volume (*dome>GFP*); number of lymph glands analysed (n) = 20. Right, graphic representation of the mitotic cell distribution in Col-positive and -negative progenitors in early (EL3) and mid (ML3) third instar larvae. In EL3 LGs, 88 ± 8% are within the *dome>GFP*/Col co-expression domain (red), the other cells (green) are *dome>GFP* positive. In ML3 LGs, 58 ± 6% are within the Col expressing domain (red). Vertical bars correspond to SD. Scale bars: 40 μm.

In wild type (wt) second larval instar lymph glands, both *in situ* hybridization and immuno-staining indicated that all cells of the anterior lobes express low levels of *col* (Fig 1A, 1E and 1E’), in addition to high levels in the PSC and stochastic expression in the secondary lobes [10]. At this stage, primary lobe cells expressed *dome-Gal4>mCD8-GFP (dome>GFP)*, a reporter insertion in *domeless*, which encodes the receptor of the JAK/STAT signalling pathway (Fig 1E) [5, 11]. In early third instar larvae, when hemocyte differentiation starts to form a cortical zone (CZ), both *col* mRNA and Col protein become restricted to the innermost part of the *dome>GFP* population of progenitors (Fig 1B and 1H), which displays a pseudo-epithelial structure [5]. In mid third instar larval lymph gland, *col* expression in the medullary zone (called *col*^*MZ*^ hereafter) overlaps with the expression domain of the thioester-containing protein 4 *(tep4)* (Fig 1F and 1F’) [36], which marks a sub-set of hemocyte progenitors in the medullary zone (Fig 1G and 1G’). In late third instar larvae, very low levels of *col* expression could be detected in medullary zone cells (Fig 1D). In order to better characterize *col*^*MZ*^ progenitors, we examined the cell division pattern in early and mid third instar larval lymph glands, by co-staining for Col, *dome>GFP* and the mitotic cell marker H3P (phosphorylated histone H3). Active proliferation of *dome>GFP* progenitors in early third instar larvae was previously shown to contribute most of primary lobe growth [12, 15]. In early third instar larval lymph gland, we found that the *col*^*MZ*^ domain concentrates 88% of the H3P positive cells, while representing only 38% of the total volume of the medullary zone (Fig 1I). This distribution showed that *col*^*MZ*^ progenitors actively divide in early third instar larval lymph gland. In mid third instar larval lymph gland, 58% of H3P positive cells are localized in the *col*^*MZ*^ domain (Fig 1I). All together, these data revealed the existence of a core population of proliferating blood cell progenitors in the third instar larval lymph gland, which co-express *col* and *tep4*.

### Col^MZ^ prevents hematopoietic progenitors from differentiating

**Fig 2:**
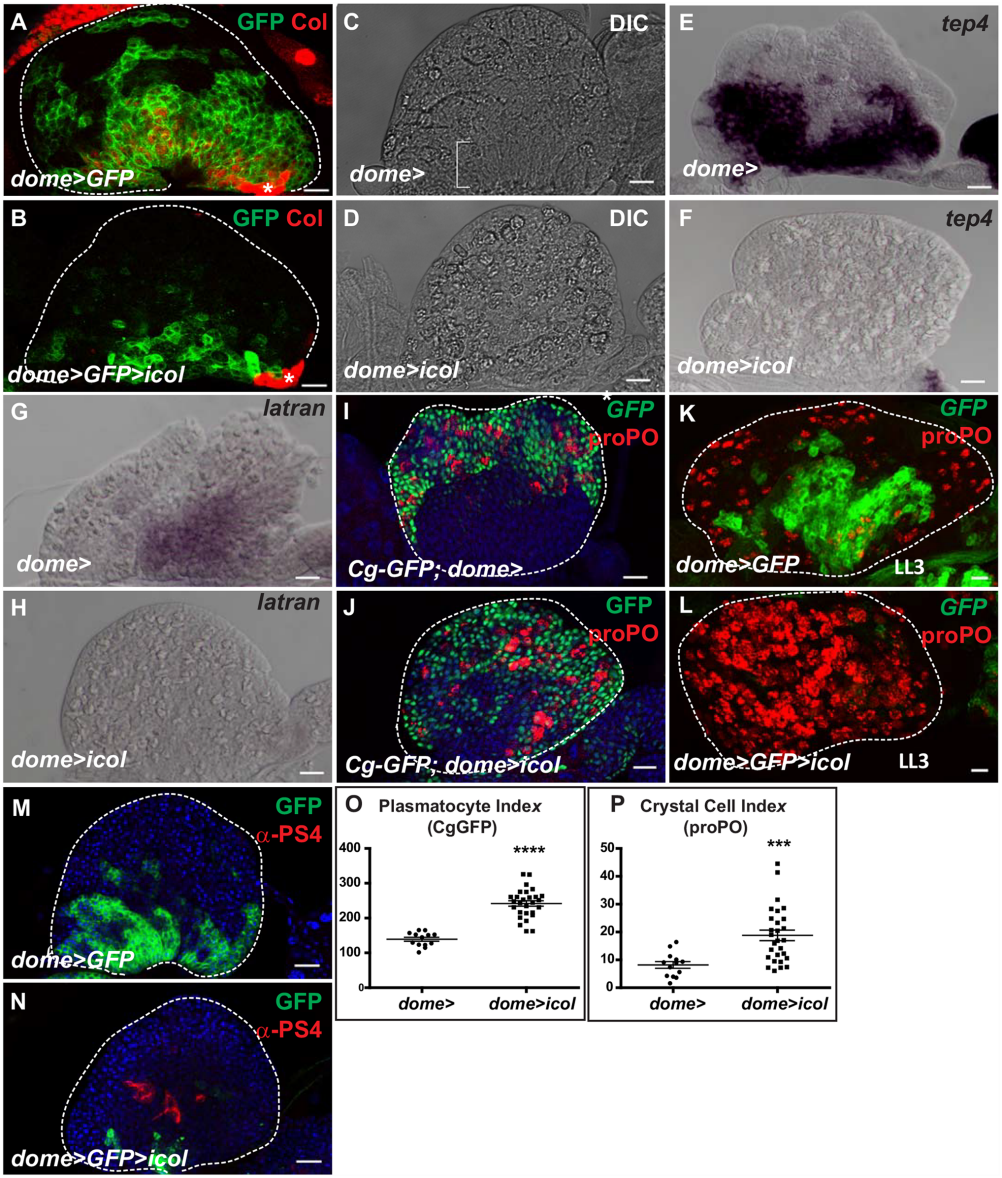
*col*^*MZ*^ maintains progenitors in the maturing lymph gland. *dome>* lymph glands at mid- (A to J, M, N) or late (K, L) third instar larvae. (A, B) Immuno-staining of control (*dome>GFP*, A) and *dome>GFP>colRNAi* (*dome>GFP>icol*) lymph glands (B), for GFP (green) and Col (red), single confocal sections. The PSC is indicated by an asterisk. (C, D) Examination of lymph glands by differential interference contrast (DIC) microscopy, showing the loss of epithelial-like organization of the medullary zone (bracket in C) in *dome>icol* lymph glands (D). (E-H) *in situ* hybridization to *tep4* (E, F) and *latran* (G, H) mRNA in wild type (E, G) and *dome>icol* (F, H) lymph glands. (I-L) Staining of mid- (I, J) and late third instar lymph gland (K, L) for Cg-GFP (I, J, green) and proPO (I- L, red) to visualize plasmatocytes and crystal cells respectively, or for GFP (K, L) to visualize medullary zone progenitors. Stacks of entire primary lobes are shown. (M, N) Staining of *dome>GFP* (M) and *dome>GFP>icol* lymph glands (N) for GFP (green) and the lamellocyte marker α-PS4 (red), single confocal sections. (O, P) Plasmatocyte (Cg-GFP) and crystal cell (proPO) indexes (number per primary lobe volume) in early third instar larval lymph gland anterior lobes. For all quantifications presented: error bars represent SEM and *P<0.1; **P<0.01; ***P<0.001; ****P<0.0001 and ns (not significant); Student’s t test. Scale bars: 40 μm. TOPRO3 (blue in I, J, M, N).

In order to address *col* function in the medullary zone, we examined progenitor and differentiated hemocyte populations when *col* expression was knocked down specifically in medullary zone cells, using RNA interference (*dome>icol*). In third instar larvae, we found that the population of *dome>GFP* cells decreased prematurely in *dome>icol* lymph glands (Fig 2A and 2B), with only a few GFP-positive cells persisting up to the end of larval development (Fig 2K and 2L). In parallel to the loss of GFP expression, *col* knock down in the medullary zone lead to the loss of the medullary zone epithelial-like structure (Fig 2C and 2D). Furthermore, the expression domain of two progenitor cell markers, *latran (lat)/eye transformer (et)* and *tep4*, was drastically reduced (Fig 2E–2H), supporting the conclusion that *col*^*MZ*^ expression is required for progenitor identity.

To determine whether the loss of progenitor markers and medullary zone shaping when *col* is knocked down in the medullary zone impacted hemocyte differentiation, we stained *dome>icol* lymph glands for plasmatocyte and crystal cell markers, Cg25C-GFP [30] and proPO [11], respectively. In wild type third instar lymph gland, expression of these markers was restricted to the cortical zone (Fig 2I). By contrast, when *col* was knocked down in the medullary zone, the innermost part of the anterior lobes already showed signs of differentiation at mid third larval instar (Fig 2J). Quantification of differentiated hemocytes in anterior lobes revealed a two-fold increase in both plasmatocyte and crystal cell numbers in early third larval instar when *col* expression was knocked down in the medullary zone compared to wild type (Fig 2O and 2P; S1C and S1D Fig). In late third instar larval lymph glands, the bi-zonal organization of the anterior lobes was lost and differentiated hemocytes populated the entire anterior lobes, at the expense of progenitors (Fig 2K and 2L). However, no obvious differentiation defect could be associated in second instar larval lymph glands when *col* expression was knocked down in the medullary zone, indicating that other developmental signals are required for hemocyte differentiation (S1A, S1B and S1E–S1F’ Fig). Whereas in control lymph glands, some lamellocytes (less than 10) differentiate in few lymph glands (18%, n = 49), most lymph glands lacking *col* in the medullary zone (75%, n = 52) develop lamellocytes as in control (Fig 2M and 2N). Finally, there was no change in expression of either signaling (*hhF4fGFP*) (S2A and S2B Fig) or identity (Antp, Col) markers (S2C, S2D, S1G and S1H Figs) of the PSC in *dome>icol* lymph glands, indicating that hemocyte differentiation upon removal of *col* in the medullary zone is not a secondary consequence of a PSC identity defect. Together, these data revealed a previously unnoticed, cell-autonomous function of *col* in blood cell progenitors in preserving their identity throughout third instar.

### Col^MZ^ and the PSC independently regulate distinct facets of lymph gland homeostasis

**Fig 3:**
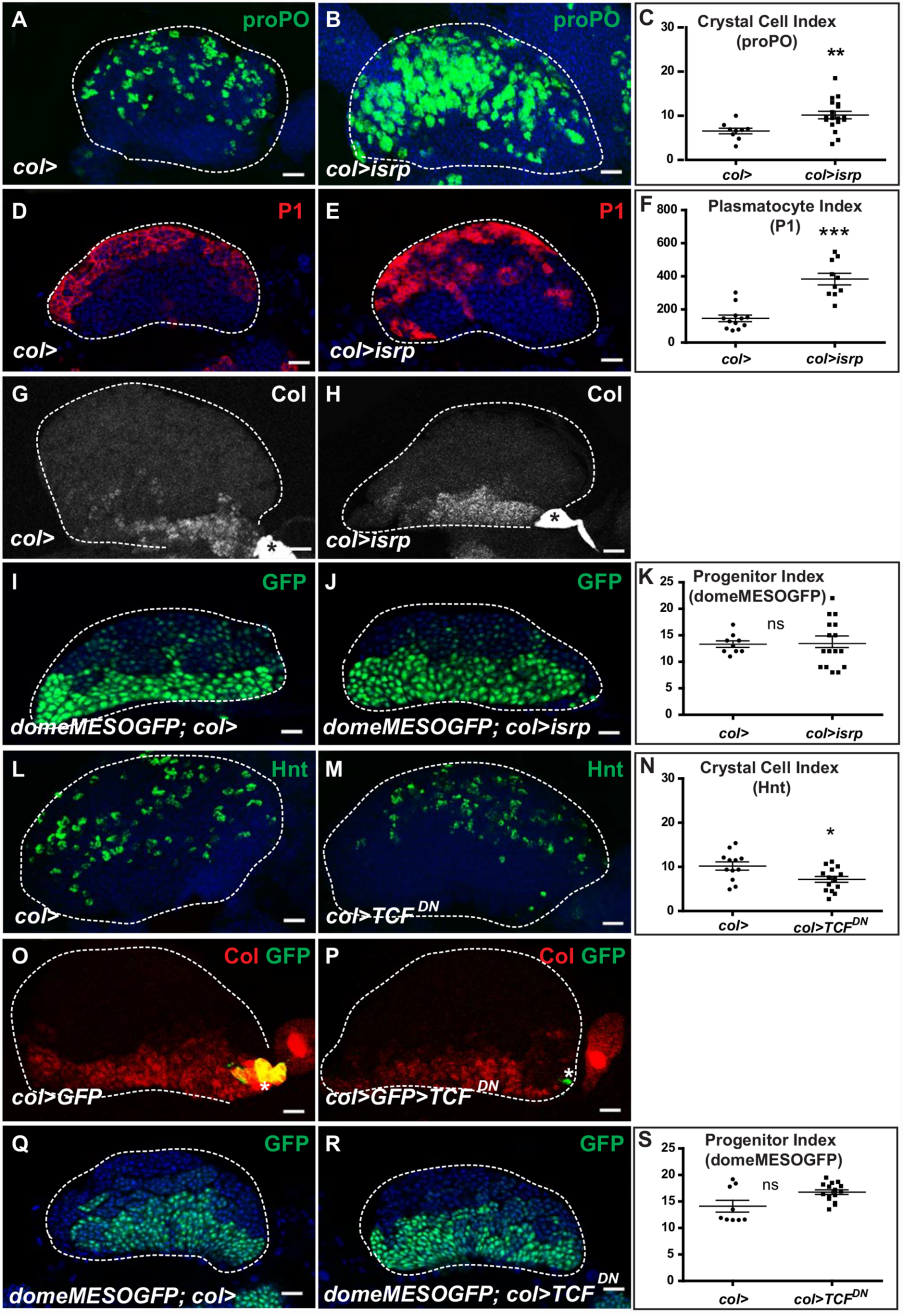
Modifying PSC cell number and signaling does not affect progenitor maintenance. Confocal views are z stacks of mid third instar larval lymph glands, except in (I, J, Q, R) which are middle sections of z stacks. (A-E) Immunostaining of control (A, D) and p*col-Gal4>srp* (*col>srp*) lymph glands (B, E), for proPO (green, A, B) and P1 (red, D, E), showing increased hemocyte differentiation upon specific knock down of *srp* in PSC cells (B, E). (C, F) Plasmatocyte and crystal cell indexes (number per primary lobe volume). (G- J) Col immunostaining (white, G, H) and *domeMESOGFP* expression (green, I, J) showing that progenitors are maintained in *col>srp* lymph glands. (K) *domeMESOGFP*-positive progenitor index (volume of GFP-positive cells per primary lobe volume). (L-M) Immunostaining of control, (L) and *col>TCF*^*DN*^ lymph glands (M) for Hnt (green), a crystal cell marker. (N) Crystal cell index (number of crystal cells per primary lobe volume) in control and *col>TCF*^*DN*^ lymph glands. Immunostaining for Col (red, O, P) and *domeMESOGFP* (green, Q, R). (S) *domeMESOGFP* progenitor index (volume of GFP-positive cells per primary lobe volume) in control and *col>TCF*^*DN*^ lymph glands. Scale bars: 40 μm. TOPRO3 (blue) marks nuclei.

Enhanced hemocyte differentiation was previously reported in both *col* mutant larvae [11] and *hh*^*ts*^ mutants [13], and interpreted as a consequence of PSC loss or of impaired *hh* expression in PSC cells, respectively. Enhanced hemocyte differentiation reported here upon *col* depletion in the medullary zone raised the question of whether *col*^*MZ*^ expression was controlled by Hh issued from the PSC. The GATA factor Serpent (Srp) controls *hh* expression in the PSC [31]. We therefore suppressed *hh* expression in PSC cells by downregulating *srp* using the PSC driver *pcol-Gal4 (col>isrp)*. In agreement with previous reports [13, 31], we confirmed that loss of *hh* expression in the PSC, visualized by the *hh* transcriptional reporter *hhF4fGFP* [31] (S3A and S3B Fig), caused a significant increase in crystal cell and plasmatocyte differentiation compared to wild type, confirming that the PSC regulates the rate of hemocyte differentiation (Fig 3A–3F). However, increased hemocyte differentiation remained restricted to the periphery of the lymph gland, unlike in lymph glands lacking *col* in the medullary zone (compare Figs 3B with 2J and 2L). We then looked at expression of both *col* in the medullary zone (Fig 3G and 3H) and *domeMESOGFP*, a marker of JAK/STAT positive-progenitors, (Fig 3I–3K) and found that neither was lost in third instar *col>isrp* lymph glands. Altogether, our data indicate that *col* expression in the medullary zone and its function in those cells are independent of *hh* expressed by PSC cells.

To further investigate a possible regulation of *col*^*MZ*^ expression by the PSC, we genetically modified the PSC size. PSC cell number was reduced from an average of 40 to less than 5 cells per lobe by antagonizing Wg signaling in the PSC, through expression of a dominant-negative form of TCF*/pangolin(pan)* (*col>TCF*^*DN*^) (S3E Fig) [20, 21, 26]. Quantification of cell differentiation in this genetic context revealed a reproducible, mild reduction of crystal cell number in anterior lobes (Fig 3L–3N). In contrast, neither *col*^*MZ*^ (Fig 3O and 3P) nor *domeMESOGFP* expression (Fig 3Q–3S) was detectably modified. In a reciprocal experiment, we overexpressed the proto-oncogene Myc in PSC cells (*col>Myc*), which leads to strong increase of both the PSC cell number and the pool of *domeMESO*-positive medullary zone progenitors in anterior lobes, at the expense of differentiated hemocytes [20]. We found that *col*^*MZ*^ expression was not noticeably modified in this background (S3C and S3D Fig). Thus, altering PSC cell number, and thereby overall PSC signaling, does not significantly impact *col*^*MZ*^ expression. From these data, we conclude that *col*^*MZ*^ expression is independent of the PSC, and that *col* expressed in the medullary zone and in the PSC control blood cell homeostasis in the lymph gland, by regulating progenitor maintenance and hemocyte differentiation, respectively.

### Switching off *col* expression in the medullary zone upon parasitism is independent of the PSC

**Fig 4:**
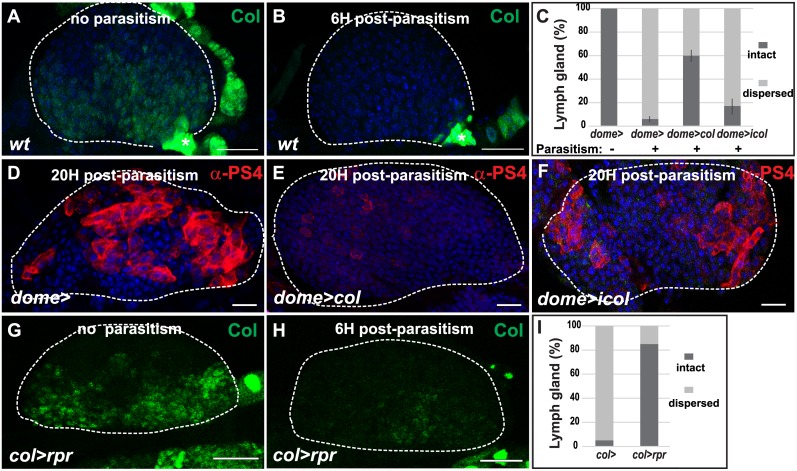
Down regulation of *col* expression in the medullary zone upon parasitism is independent of the PSC. Single confocal sections are shown. (A, B) Immunostaining of early third instar larval lymph glands 6H post wasp parasitism, showed that Col expression (green) is lost in the medullary zone but not the PSC (asterisk). (C) Percentage of dispersed lymph glands 24H post-parasitism. Vertical bars correspond to SD. The number of lymph glands analyzed is: n = 15 (*dome*>; no parasitism); n = 33 (*dome*>; parasitism); n = 17 (*dome>col*; parasitism); n = 29 (*dome>icol*; parasitism). (D-F) anti-αPS4 immunostaining, showing massive differentiation of lamellocytes 20H post-parasitism in both control (D) and *dome>icol* lymph glands (F). (E) Conversely, few lamellocytes differentiate when Col expression is maintained in the medullary zone (*dome>col*). (G-I) Ablation of PSC cells (*col>rpr*) severely reduces lymph gland dispersal 24H post-parasitism (I), without impeding *col*^*MZ*^ switching off 6H post-parasitism (H). Scale bars: 40 μm. TOPRO3 (blue).

Wasp parasitism provokes a phase of active proliferation in early third instar larval lymph glands, followed by massive lamellocyte differentiation, at the expense of progenitor maintenance [11, 12]. We found that *col*^*MZ*^ expression was specifically lost 6H post-parasitism, while *col* remained strongly expressed in PSC cells (Fig 4A and 4B) [10, 28]. This observation raised the possibility that silencing *col*^*MZ*^ activity was a pre-requisite for massive lamellocyte differentiation. To test this possibility, we artificially forced *col* expression in the medullary zone (*dome>col*), in larvae that were wasp parasitized at early third instar. While dispersal of lymph glands and release of lamellocytes into circulation was robustly observed in wild type larvae 24H post-parasitism, it was only observed in 40% of *dome>col* lymph glands parasitized larvae (Fig 4C). Furthermore, only few differentiated lamellocytes were observed at the cortex of *dome>col* lymph gland anterior lobes which did not disperse 24H post-parasitism (Fig 4D and 4E). We conclude that down-regulation of *col* expression in the medullary zone following wasp infestation is a pre-requisite for efficient differentiation of lamellocytes in the lymph gland and their release into the hemolymph upon lymph gland dispersal.

The question remained as to whether *col* expression in the medullary zone endowed progenitor cells with the ability to differentiate into lamellocytes. To address this question, we subjected to wasp parasitism larvae in which *col*^*MZ*^ was downregulated from second instar larval stage (*dome>icol*), and analyzed lymph glands 24H post-parasitism. We found that *col* knock down in the medullary zone lead to lymph gland dispersal, similar to wild type (Fig 4C). Staining with anti-αPS4, a lamellocyte-specific marker [11], confirmed that lamellocytes differentiated massively in the lymph gland as in wild type (Fig 4D and 4F). Thus, *col*^MZ^ expression is not required for cell fate specification of blood cell progenitors into lamellocytes upon parasitism.

Our previous studies identified the PSC as essential for lymph gland responsiveness to parasitism [10, 11]. Indeed, massive differentiation of lamellocytes did not occur in *col* mutant lymph glands where the PSC was not specified [10]. Neither did it occur in wild type larvae where PSC cells were ablated by targeted expression of reaper (*rpr*), a cell-death inducer [11]. Our finding that *col*^*MZ*^ was downregulated in response to parasitism thus opened the possibility that this regulation was dependent on PSC function. To test this hypothesis, we eliminated PSC cells (*col>rpr*) in larvae (S4A and S4B Fig), which were then subjected to parasitism before examining lymph glands. Although lymph glands lacking a PSC rarely dispersed 24H post-parasitism (Fig 4I), *col*^*MZ*^ was notably downregulated 6H post-parasitism in *col>rpr* lymph glands, similar to wild type (Fig 4G and 4H). Thus, the down regulation of *col*^*MZ*^ expression in blood cell progenitors upon parasitism does not require the PSC. Moreover, these data indicate that progenitor cells respond directly to immune cues.

## Discussion

Here, we characterize a novel core population of blood cell progenitors in the *Drosophila* lymph gland that expresses *col*, where it plays an essential, cell-autonomous function in preventing hematopoietic progenitors from differentiating. The same conclusion was reported recently by another team, using similar experiments [37]. Maintenance of progenitors was previously assigned to *col* expression in the PSC [11, 13]. Here, we further redefine the PSC function in normal developmental conditions, which is to regulate the rate of blood cell differentiation, and we confirm its requirement for lamellocytes production and lymph gland dispersal upon wasp parasitism. We further show that switching off *col* expression in the medullary zone is also required for lymph gland response to parasitism, independently from PSC activity. Thus, two independent Col expression sites in the lymph gland, in core hematopoietic progenitors and the PSC, control *Drosophila* larval hematopoiesis under normal developmental conditions and upon wasp parasitism, thereby defining a new lymph gland homeostasis paradigm.

### *col* expression in the medullary zone defines a sub-population of lymph gland proliferating progenitors

Our data confirm that, in addition to the PSC and posterior lobes, *col* is expressed in a sub-population of progenitors in the medullary zone [10, 37]. Hence, the medullary zone population is heterogeneous, as it contains both *col*-positive and -negative hematopoietic progenitor cells. In addition, we show that progenitors expressing Col actively proliferate and could contribute to the extensive lymph gland growth that occurs in early third instar larvae, while outer medullary zone, *col*-negative cells could correspond to intermediate progenitors primed to differentiate and contributing to hemocyte differentiation along lymph gland maturation. The persistence of progenitors expressing *col* in anterior lobes up to metamorphosis raises the question of their fate beyond larval development. We have previously shown that *col* was expressed in a fraction of cells in posterior lymph gland lobes that do not show signs of hemocyte differentiation in normal developmental conditions [10], and we proposed that posterior lobes could be reservoirs of precursors for adult hemocytes [38], one possibility recently supported by lineage tracing experiments [39]. Our finding of a pool of *col*-expressing progenitors in the medullary zone raises the question of the respective contributions of these and posterior lobe cells to adult hematopoiesis.

We previously showed that forced *col* expression in the entire medullary zone prevented hemocyte differentiation [11]. This study, in addition to data presented by Benmimoun et al. (2015), indicating that *col* expression in the medullary zone maintains progenitors in third instar lymph glands, shows that the restriction of *col* expression to a core medullary zone progenitors that occurs in early third instar larvae allows Col negative progenitors to progress towards differentiation. In addition, our study shows that forcing *col* expression in progenitors prevents them from differentiating massively into lamellocytes in response to parasitism. This finding disagrees with the conclusion raised by Benmimoun et al. (2015) that Col overexpression does not affect lamellocyte differentiation in response to wasp parasitism although the extent of differentiation was not quantified. While varying levels of ectopic *col* expression in progenitors could possibly explain this discrepancy, the notable defect of lymph glands dispersal upon parasitism strengthens the conclusion that removal of *col* expression in the medullary zone is required for massive lamellocyte differentiation. Various genes and signalling pathways have been shown to regulate progenitor cell maintenance versus differentiation in the medullary zone [2, 13, 16, 18, 19, 21, 23, 25, 31, 40–49]. Integrating the regulation of *col* expression in the medullary zone into regulatory networks controlling blood cell homeostasis is an important line of future investigation.

### Contrasting roles of the PSC under normal and parasitism conditions

Modifying the PSC cell number and/or gene expression program showed that the PSC, while not required to maintain *col*^*MZ*^ progenitors, regulates hemocyte differentiation. Reducing *srp* expression, thereby *hh* production, in PSC cells favors crystal cell and plasmatocyte differentiation (Fig 3) [13, 16]. Conversely, increasing levels of PSC signals by genetically increasing cell numbers reduce hemocyte differentiation [20, 21, 23, 24, 26, 37]. Paradoxically, reducing drastically PSC cell number can also result in decreased crystal cell differentiation without affecting blood cell progenitor maintenance (Fig 3). Reduced crystal cell number with no progenitor maintenance defect was also reported in PSC-ablated lymph glands [37]. This data supports our conclusion that the PSC regulate hemocyte differentiation in the lymph gland. How can one reconcile this apparent paradox? We hypothesize that hemocyte homeostasis integrates positive and negative differentiation signals issued from the PSC. Altering one signal at a time could thus affect PSC cell number and/or signaling ability, in the same or opposite ways. One example is reducing *col* expression in the PSC specifically in larvae, which both increases PSC cell proliferation and decreases *hh* signalling with no or little consequence on hemocyte differentiation [20]. How can the PSC regulate blood cell differentiation independently of progenitor maintenance? Many cells in third instar larval lymph glands show neither high levels of medullary zone gene expression nor expression of hemocyte differentiation genes, suggesting that they most likely represent an heterogeneous cell population encompassing intermediate, fate-restricted progenitors and precursors committed to differentiate [15, 16, 42, 50]. One attractive hypothesis is that PSC signaling regulates the differentiation of this overlooked pool of intermediate lymph gland cells, a subject of future investigation.

The PSC plays an essential role in mounting a cellular immune response to wasp parasitism (this work and [10, 11, 27, 37]). Our present data further establish that silencing *col* expression in the medullary zone is also essential for this response, independently from PSC activity (Fig 4). Thus, our study establishes, for the first time, that several signals, PSC-relayed and PSC-independent, must be integrated by medullary zone cells for an effective immune response, pointing to a more integrated process than previously thought for fighting wasp parasitism. A previous study showed that switching off JAK/STAT signalling in medullary zone cells was necessary for massive lamellocyte production upon wasp parasitism [34], suggesting cross-talks between JAK/STAT signalling and the regulation of *col* expression in the medullary zone.

### Conservation of Col/EBF functions during hematopoiesis

Four mammalian Col orthologs, EBF1-4 have been characterized [51]. EBF1, initially characterized for its role in immature B-Cell progenitors [52], regulates differentiation, stage-specific signaling, proliferation, and survival of B cells [53]. Mouse EBF2 was later shown to be expressed in immature osteoblasts, one component of the endosteal HSC niche in the adult bone marrow [54]. Thus, different EBFs control different aspects of vertebrate hematopoiesis and immune cell differentiation. The different roles of Col in the *Drosophila* lymph gland provide a framework for investigating the evolution of COE (Collier/EBF) hematopoietic functions throughout the animal kingdom.

## Supporting Information

S1 Fig *col* knock down in the medullary zone does not trigger differentiation of hematopoietic progenitors until early third instar larval stage. *dome>GFP* lymph glands at second (L2) (A, B, E-F’) or early (EL3) (C, D) third instar larvae. (A, F) Immunostaining of control (*dome>GFP*, A, C, E) and (*dome>GFP>icol*, B, D, F) lymph glands for GFP (green), P1 (red, A-D), and Col (red, E-F; white, E’-F’). Col expression in the PSC is unaffected (asterisk in E, F). (G, H) *col* expression in the medullary zone is lost upon *dome>icol* expression in third instar larval lymph glands. Scale bars: 40 μm. TOPRO3 (blue in A-F; G-H).
(EPS)

S2 Fig *col* knock down in the medullary zone does not affect the expression of PSC-specific genes. *hhF4fGFP* (green in A, B) and Antp (red in C, D) expression in the PSC (asterisk) is not affected in *dome>icol* third instar larval lymph glands, in contrast to increasing plasmatocyte (Cg-GFP, green in C, D) and crystal cell (proPO, red and arrowhead in C, D) differentiation. Scale bars: 40 μm. TOPRO3 (blue).
(EPS)

S3 Fig Modifying either PSC cell number or signaling does not affect *col*^*MZ*^ expression. (A, B) Loss of *hh* expression (*hhF4fGFP*, green) in the PSC (Col immunostaining, red) in *col>isrp* lymph glands. (C, D) Drastic increase in the number of PSC cells (*hhF4fGFP*, green, asterisk) is observed when Myc is overexpressed in the PSC (*col>Myc*), while *col* expression in the medullary zone is not affected (red). Scale bars: 40 μm. TOPRO3 (blue). (E) Quantification of PSC cell number per anterior lobe volume using the PSC-specific marker Antp.
(EPS)

S4 Fig *reaper* (*rpr*) expression in PSC cells leads to their loss. (A-B) Immuno-staining of *col>GFP* (control, A) and *col>GFP>rpr* (B) early third instar larval lymph glands, for GFP (green) and Col (red). Note the lack of PSC cells (asterisk, A) in *col>GFP>rpr* lymph glands (B). Pericardiac cells (p.c), posterior/secondary lobes (p.l) are indicated. Scale bars: 40 μm.
(EPS)

## References

[1] Crozatier M, Krzemien J, Vincent A. The hematopoietic niche: a Drosophila model, at last. Cell Cycle. 2007;6(12):1443–4. . 17582220

[2] Crozatier M, Vincent A. Drosophila: a model for studying genetic and molecular aspects of haematopoiesis and associated leukaemias. Dis Model Mech. 2011;4(4):439–45. 10.1242/dmm.007351 21669932 PMC3124048

[3] Fossett N. Signal transduction pathways, intrinsic regulators, and the control of cell fate choice. Biochim Biophys Acta. 2013;1830(2):2375–84. 10.1016/j.bbagen.2012.06.005 22705942 PMC3477240

[4] Gold KS, Brückner K. Drosophila as a model for the two myeloid blood cell systems in vertebrates. Exp Hematol. 2014;42(8):717–27. 10.1016/j.exphem.2014.06.002 . 24946019 PMC5013032

[5] Jung SH, Evans CJ, Uemura C, Banerjee U. The Drosophila lymph gland as a developmental model of hematopoiesis. Development. 2005;132(11):2521–33. 10.1242/dev.01837 . 15857916

[6] Martinez-Agosto JA, Mikkola HK, Hartenstein V, Banerjee U. The hematopoietic stem cell and its niche: a comparative view. Genes Dev. 2007;21(23):3044–60. 10.1101/gad.1602607 . 18056420

[7] Minakhina S, Steward R. Hematopoietic stem cells in Drosophila. Development. 2010;137(1):27–31. 10.1242/dev.043943 20023157 PMC2796932

[8] Lanot R, Zachary D, Holder F, Meister M. Postembryonic hematopoiesis in Drosophila. Dev Biol. 2001;230(2):243–57. 10.1006/dbio.2000.0123 . 11161576

[9] Grigorian M, Mandal L, Hartenstein V. Hematopoiesis at the onset of metamorphosis: terminal differentiation and dissociation of the Drosophila lymph gland. Dev Genes Evol. 2011;221(3):121–31. 10.1007/s00427-011-0364-6 21509534 PMC4278756

[10] Crozatier M, Ubeda JM, Vincent A, Meister M. Cellular immune response to parasitization in Drosophila requires the EBF orthologue collier. PLoS Biol. 2004;2(8):E196 10.1371/journal.pbio.0020196 15314643 PMC509289

[11] Krzemień J, Dubois L, Makki R, Meister M, Vincent A, Crozatier M. Control of blood cell homeostasis in Drosophila larvae by the posterior signalling centre. Nature. 2007;446(7133):325–8. 10.1038/nature05650 . 17361184

[12] Sorrentino RP, Carton Y, Govind S. Cellular immune response to parasite infection in the Drosophila lymph gland is developmentally regulated. Dev Biol. 2002;243(1):65–80. 10.1006/dbio.2001.0542 . 11846478

[13] Mandal L, Martinez-Agosto JA, Evans CJ, Hartenstein V, Banerjee U. A Hedgehog- and Antennapedia-dependent niche maintains Drosophila haematopoietic precursors. Nature. 2007;446(7133):320–4. 10.1038/nature05585 17361183 PMC2807630

[14] Mandal L, Banerjee U, Hartenstein V. Evidence for a fruit fly hemangioblast and similarities between lymph-gland hematopoiesis in fruit fly and mammal aorta-gonadal-mesonephros mesoderm. Nat Genet. 2004;36(9):1019–23. 10.1038/ng1404 . 15286786

[15] Krzemien J, Oyallon J, Crozatier M, Vincent A. Hematopoietic progenitors and hemocyte lineages in the Drosophila lymph gland. Dev Biol. 2010;346(2):310–9. 10.1016/j.ydbio.2010.08.003 . 20707995

[16] Tokusumi T, Tokusumi Y, Hopkins DW, Shoue DA, Corona L, Schulz RA. Germ line differentiation factor Bag of Marbles is a regulator of hematopoietic progenitor maintenance during Drosophila hematopoiesis. Development. 2011;138(18):3879–84. 10.1242/dev.069336 21813570 PMC3160086

[17] Lebestky T, Jung SH, Banerjee U. A Serrate-expressing signaling center controls Drosophila hematopoiesis. Genes Dev. 2003;17(3):348–53. 10.1101/gad.1052803 12569125 PMC195988

[18] Mondal BC, Mukherjee T, Mandal L, Evans CJ, Sinenko SA, Martinez-Agosto JA, et al Interaction between differentiating cell- and niche-derived signals in hematopoietic progenitor maintenance. Cell. 2011;147(7):1589–600. 10.1016/j.cell.2011.11.041 22196733 PMC4403793

[19] Mondal BC, Shim J, Evans CJ, Banerjee U. Pvr expression regulators in equilibrium signal control and maintenance of Drosophila blood progenitors. Elife. 2014;3:e03626 10.7554/eLife.03626 25201876 PMC4185420

[20] Pennetier D, Oyallon J, Morin-Poulard I, Dejean S, Vincent A, Crozatier M. Size control of the Drosophila hematopoietic niche by bone morphogenetic protein signaling reveals parallels with mammals. Proc Natl Acad Sci U S A. 2012;109(9):3389–94. 10.1073/pnas.1109407109 22331866 PMC3295293

[21] Sinenko SA, Mandal L, Martinez-Agosto JA, Banerjee U. Dual role of wingless signaling in stem-like hematopoietic precursor maintenance in Drosophila. Dev Cell. 2009;16(5):756–63. 10.1016/j.devcel.2009.03.003 19460351 PMC2718753

[22] Khadilkar RJ, Rodrigues D, Mote RD, Sinha AR, Kulkarni V, Magadi SS, et al ARF1-GTP regulates Asrij to provide endocytic control of Drosophila blood cell homeostasis. Proc Natl Acad Sci U S A. 2014;111(13):4898–903. 10.1073/pnas.1303559111 24707047 PMC3977295

[23] Benmimoun B, Polesello C, Waltzer L, Haenlin M. Dual role for Insulin/TOR signaling in the control of hematopoietic progenitor maintenance in Drosophila. Development. 2012;139(10):1713–7. 10.1242/dev.080259 . 22510984

[24] Tokusumi T, Tokusumi Y, Hopkins DW, Schulz RA. Bag of Marbles controls the size and organization of the Drosophila hematopoietic niche through interactions with the Insulin-like growth factor pathway and Retinoblastoma-family protein. Development. 2015;142(13):2261–7. 10.1242/dev.121798 . 26041767

[25] Tokusumi Y, Tokusumi T, Shoue DA, Schulz RA. Gene regulatory networks controlling hematopoietic progenitor niche cell production and differentiation in the Drosophila lymph gland. PLoS One. 2012;7(7):e41604 10.1371/journal.pone.0041604 22911822 PMC3404040

[26] Lam V, Tokusumi T, Tokusumi Y, Schulz RA. bantam miRNA is important for Drosophila blood cell homeostasis and a regulator of proliferation in the hematopoietic progenitor niche. Biochem Biophys Res Commun. 2014;453(3):467–72. 10.1016/j.bbrc.2014.09.109 . 25280996 PMC5964997

[27] Sinenko SA, Shim J, Banerjee U. Oxidative stress in the haematopoietic niche regulates the cellular immune response in Drosophila. EMBO Rep. 2012;13(1):83–9. 10.1038/embor.2011.223 22134547 PMC3246251

[28] Krzemien J. Control of larval hematopoiesis in Drosophila; microenvironment, precursors and cell lineage.: Ph.D thesis. University of Toulouse III-Paul Sabatier; 2008.

[29] Bourbon HM, Gonzy-Treboul G, Peronnet F, Alin MF, Ardourel C, Benassayag C, et al A P-insertion screen identifying novel X-linked essential genes in Drosophila. Mech Dev. 2002;110(1–2):71–83. . 11744370 10.1016/s0925-4773(01)00566-4

[30] Sorrentino RP, Tokusumi T, Schulz RA. The Friend of GATA protein U-shaped functions as a hematopoietic tumor suppressor in Drosophila. Dev Biol. 2007;311(2):311–23. 10.1016/j.ydbio.2007.08.011 . 17936744

[31] Tokusumi Y, Tokusumi T, Stoller-Conrad J, Schulz RA. Serpent, suppressor of hairless and U-shaped are crucial regulators of hedgehog niche expression and prohemocyte maintenance during Drosophila larval hematopoiesis. Development. 2010;137(21):3561–8. 10.1242/dev.053728 20876645 PMC2964091

[32] Rivas ML, Cobreros L, Zeidler MP, Hombría JC. Plasticity of Drosophila Stat DNA binding shows an evolutionary basis for Stat transcription factor preferences. EMBO Rep. 2008;9(11):1114–20. 10.1038/embor.2008.170 18802449 PMC2556238

[33] Bischof J, Maeda RK, Hediger M, Karch F, Basler K. An optimized transgenesis system for Drosophila using germ-line-specific phiC31 integrases. Proc Natl Acad Sci U S A. 2007;104(9):3312–7. 10.1073/pnas.0611511104 17360644 PMC1805588

[34] Makki R, Meister M, Pennetier D, Ubeda JM, Braun A, Daburon V, et al A short receptor downregulates JAK/STAT signalling to control the Drosophila cellular immune response. PLoS Biol. 2010;8(8):e1000441 10.1371/journal.pbio.1000441 20689801 PMC2914635

[35] Russo J, Dupas S, Frey F, Carton Y, Brehelin M. Insect immunity: early events in the encapsulation process of parasitoid (Leptopilina boulardi) eggs in resistant and susceptible strains of Drosophila. Parasitology. 1996;112 (Pt 1):135–42. . 8587797 10.1017/s0031182000065173

[36] Irving P, Ubeda JM, Doucet D, Troxler L, Lagueux M, Zachary D, et al New insights into Drosophila larval haemocyte functions through genome-wide analysis. Cell Microbiol. 2005;7(3):335–50. 10.1111/j.1462-5822.2004.00462.x . 15679837

[37] Benmimoun B, Polesello C, Haenlin M, Waltzer L. The EBF transcription factor Collier directly promotes Drosophila blood cell progenitor maintenance independently of the niche. Proc Natl Acad Sci U S A. 2015;112(29):9052–7. 10.1073/pnas.1423967112 26150488 PMC4517242

[38] Krzemien J, Crozatier M, Vincent A. Ontogeny of the Drosophila larval hematopoietic organ, hemocyte homeostasis and the dedicated cellular immune response to parasitism. Int J Dev Biol. 2010;54(6–7):1117–25. 10.1387/ijdb.093053jk . 20711989

[39] Ghosh S, Singh A, Mandal S, Mandal L. Active hematopoietic hubs in Drosophila adults generate hemocytes and contribute to immune response. Dev Cell. 2015;33(4):478–88. 10.1016/j.devcel.2015.03.014 25959225 PMC4448147

[40] Owusu-Ansah E, Banerjee U. Reactive oxygen species prime Drosophila haematopoietic progenitors for differentiation. Nature. 2009;461(7263):537–41. 10.1038/nature08313 19727075 PMC4380287

[41] Mukherjee T, Kim WS, Mandal L, Banerjee U. Interaction between Notch and Hif-alpha in development and survival of Drosophila blood cells. Science. 2011;332(6034):1210–3. 10.1126/science.1199643 21636775 PMC4412745

[42] Dragojlovic-Munther M, Martinez-Agosto JA. Multifaceted roles of PTEN and TSC orchestrate growth and differentiation of Drosophila blood progenitors. Development. 2012;139(20):3752–63. 10.1242/dev.074203 22951642 PMC3445307

[43] Dragojlovic-Munther M, Martinez-Agosto JA. Extracellular matrix-modulated Heartless signaling in Drosophila blood progenitors regulates their differentiation via a Ras/ETS/FOG pathway and target of rapamycin function. Dev Biol. 2013;384(2):313–30. 23603494 10.1016/j.ydbio.2013.04.004 PMC4256155

[44] Shim J, Gururaja-Rao S, Banerjee U. Nutritional regulation of stem and progenitor cells in Drosophila. Development. 2013;140(23):4647–56. 10.1242/dev.079087 24255094 PMC3833425

[45] Shim J, Mukherjee T, Mondal BC, Liu T, Young GC, Wijewarnasuriya DP, et al Olfactory control of blood progenitor maintenance. Cell. 2013;155(5):1141–53. 10.1016/j.cell.2013.10.032 24267893 PMC3865989

[46] Gao H, Wu X, Fossett N. Odd-skipped maintains prohemocyte potency and blocks blood cell development in Drosophila. Genesis. 2011;49(3):105–16. 10.1002/dvg.20711 21381183 PMC3773710

[47] Gao H, Wu X, Fossett N. Drosophila E-cadherin functions in hematopoietic progenitors to maintain multipotency and block differentiation. PLoS One. 2013;8(9):e74684 10.1371/journal.pone.0074684 24040319 PMC3764055

[48] Gao H, Wu X, Simon L, Fossett N. Antioxidants maintain E-cadherin levels to limit Drosophila prohemocyte differentiation. PLoS One. 2014;9(9):e107768 10.1371/journal.pone.0107768 25226030 PMC4167200

[49] Minakhina S, Tan W, Steward R. JAK/STAT and the GATA factor Pannier control hemocyte maturation and differentiation in Drosophila. Dev Biol. 2011;352(2):308–16. 10.1016/j.ydbio.2011.01.035 21295568 PMC3065540

[50] Ferguson GB, Martinez-Agosto JA. Yorkie and Scalloped signaling regulates Notch-dependent lineage specification during Drosophila hematopoiesis. Curr Biol. 2014;24(22):2665–72. 10.1016/j.cub.2014.09.081 25454586 PMC4256154

[51] Daburon V, Mella S, Plouhinec JL, Mazan S, Crozatier M, Vincent A. The metazoan history of the COE transcription factors. Selection of a variant HLH motif by mandatory inclusion of a duplicated exon in vertebrates. BMC Evol Biol. 2008;8:131 10.1186/1471-2148-8-131 18454855 PMC2394523

[52] Lin H, Grosschedl R. Failure of B-cell differentiation in mice lacking the transcription factor EBF. Nature. 1995;376(6537):263–7. 10.1038/376263a0 . 7542362

[53] Györy I, Boller S, Nechanitzky R, Mandel E, Pott S, Liu E, et al Transcription factor Ebf1 regulates differentiation stage-specific signaling, proliferation, and survival of B cells. Genes Dev. 2012;26(7):668–82. 10.1101/gad.187328.112 22431510 PMC3323878

[54] Kieslinger M, Hiechinger S, Dobreva G, Consalez GG, Grosschedl R. Early B cell factor 2 regulates hematopoietic stem cell homeostasis in a cell-nonautonomous manner. Cell Stem Cell. 2010;7(4):496–507. 10.1016/j.stem.2010.07.015 . 20887955

